# Preparation and Biotoxicity of Coal-Based Carbon Dot Nanomaterials

**DOI:** 10.3390/nano13243122

**Published:** 2023-12-12

**Authors:** Zhenzhou Tian, Jinyao Li, Yanming Miao, Jinzhi Lv

**Affiliations:** School of Life Science, Shanxi Normal University, Taiyuan 030006, China; 221112086@sxnu.edu.cn (Z.T.); jinyaooo_lee@163.com (J.L.)

**Keywords:** coal-based carbon dots, nano-biological effects, photocatalysis, ^1^O_2_, biotoxicity

## Abstract

Coal-based Carbon Dots (C-CDs) have gradually become a research focus due to the abundant raw materials and low preparation cost. Still, before coal-based carbon dots are widely used, a systematic biological toxicity study is the basis for the safe utilization of C-CDs. However, the level of toxicity and the mechanism of toxicity of C-CDs for organisms are still unclear. To ensure the safe utilization of C-CDs, the present study investigated C-CD nanomaterials as stressors to probe their biotoxic effects on plant, bacterial, and animal cells as well as the photocatalytic oxidative properties of C-CDs. The results showed that low concentrations of C-CDs could promote various growth indicators of wheat, and high concentrations of C-CDs had significant inhibitory effects on wheat growth; C-CDs had significant toxic effects on (*S. aureus*) at specific concentrations and were light-related; meanwhile, at concentrations of 1–5000 μg/mL, C-CDs were almost not toxic to HeLa cells; however, when irradiated at 365 nm, even low concentrations of C-CDs were toxic to cells by the mechanism that C-CDs could generate singlet oxygen (^1^O_2_) by photocatalytic oxidation under 365 nm excitation light, resulting in enhanced toxicity of C-CDs to cells.

## 1. Introduction

Carbon Dots (CDs), also known as carbon quantum dots or carbon nanodots, consist of ultrafine, dispersed, quasi-spherical carbon nanoparticles with sizes below 10 nm [1], which have easy functionalization, water solubility, good biocompatibility, and excellent photoluminescence [2,3,4,5,6]. CDs may replace metal quantum dots in various fields [7], such as biosensing [8], bioimaging [9], photocatalysis [10], and drug targeting [11], and their synthesis methods are diverse and easy to mass-produce [12,13,14].

Coal-based carbon dots (C-CDs) are an essential class of CDs. Coal, a vital fossil fuel consisting of aromatic rings and aliphatic chains, has attracted the attention of CD researchers because of its low price, abundant resources, and high carbon content. Various coal-based materials, including coal, coke, coal tar, and coal pitch, have been widely used for synthesizing CDs, and different preparation methods have been developed [15,16,17,18]. Among them, H_2_O_2_ provides a milder and more straightforward method for the oxidation of coal, which has gradually become the mainstream method for the preparation of C-CDs due to its composition of only H and O atoms, as well as its easy decomposition at high temperatures and the easy completion of the separation and purification of C-CDs from the reaction solution. Saikia et al. [17] reported a combined H_2_O_2_ oxidation and ultrasonication strategy to treat Pennsylvania anthracite and Kentucky bituminous coal. The resulting slurry was neutralized with ammonia and purified by ultrafiltration and rotary evaporation to collect a clarified C-CD liquid, which showed bright blue fluorescence under UV light at 365 nm. The selective oxidation of coals by H_2_O_2_ was initially proposed by Hu et al. [18]. The process involved a reaction in 30% H_2_O_2_ at 80 °C and centrifugation to obtain a dark yellow C-CD suspension. Due to the many potential applications of C-CDs in various biological fields, the biosafety of CDs has been widely discussed. The biosafety of C-CDs has received much attention. However, studies on the ecological impact of C-CDs are still limited, and systematic biotoxicity studies on C-CDs before they are widely used are necessary for the safe utilization of coal-based carbon sites.

C-CDs directly affect the normal physiological activities of plants, animals, and microorganisms due to their inherent physicochemical properties (e.g., different sizes and different surface modifications). Generally, C-CDs are rich in oxygen-containing groups, such as carboxyl, amino, hydroxyl, and epoxy groups. These functional groups exhibit excellent hydrophilicity and produce different surface properties in CD suspensions due to the ionization of varying surface groups, which may have further adverse effects on other organisms but needs more investigation. In addition, many applications of CDs often involve light (bioimaging, light-emitting diodes, photovoltaic cells, etc.) [19]. Under light irradiation, CDs can generate reactive oxygen species (ROS), which is an important inducer of the toxicity that CDs can produce [20,21,22,23], so studying the generation and potential biological toxicity is also an essential aspect of evaluating the toxicity of C-CDs.

**Figure 1:**
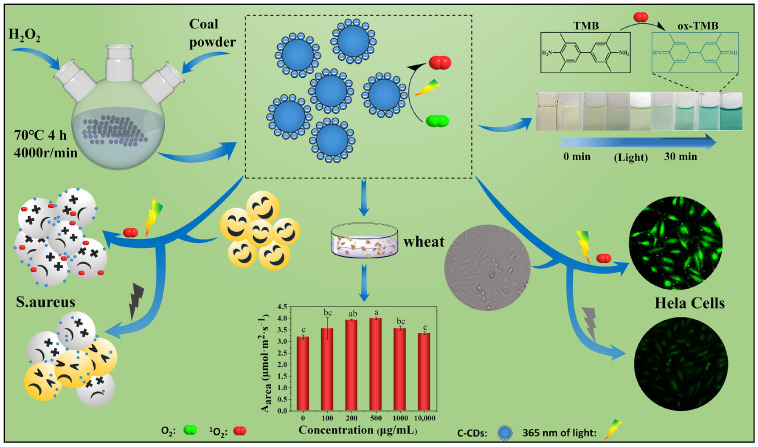
Experimental design. Different letters indicate significant differences (*p* < 0.05).

This study prepared C-CDs by oxidation with 30% hydrogen peroxide (70 °C, 4 h) using coal dust as the carbon source. The biotoxic effects of C-CDs on the plant, bacterial, and animal cells were systematically investigated using coal-based carbon dot (C-CD) nanomaterials as stress factors and wheat seeds, wheat seedlings, *S. aureus*, and HeLa cells as stress objects (Figure 1) to provide the theoretical and experimental basis for the safe utilization of C-CDs.

## 2. Materials and Methods

### 2.1. Experimental Instruments

The morphological structures of C-CDs were characterized on a JEM-2100 transmission electron microscope (Nippon Electron, Tokyo, Japan), and XPS analysis was performed on a Vario EL cube X-ray Photoelectron Spectrometer (Bruker, Karlsruhe, Germany). Fluorescence, phosphorescence, and phosphorescence lifetimes were determined by a Cary Eclipse fluorescence spectrophotometer (Agilent Technologies, Santa Clara, CA, USA); A UV-1800PC UV spectrophotometer measured ultraviolet/visible absorption spectra (UV/Vis) (Shanghai Meprotech Instruments Co., Ltd., Shanghai, China); ^1^H NMR was measured on an AVANCE III HD600 MHz NMR spectrometer (Bruker, Karlsruhe, Germany); and IR spectra were measured by a Varian 660 Fourier transform infrared spectrometer (Varian, Wilmington, DE, USA) for determination. The photosynthetic rate was measured by the Photosynthesis Measurement System (Gene, Li-6400, Lincoln, NE, USA), and the leaf area was measured by a leaf area meter (Gene, Li-3000A, Lincoln, NE, USA). The instruments related to the cell experiments included the following: MCO-15AC CO_2_ constant-temperature incubator (SANYO, Osaka, Japan), RGX-280F artificial climate incubator (Lichen, Shanghai, China), ZD-85 dual-function air-bath thermostatic shaker (Senya, Jinan, China), and ECLIPSE Ts2 inverted microscope (Nikon, Tokyo, Japan).

### 2.2. Materials

The coal dust was obtained from Datong City, China. The 30% hydrogen peroxide was purchased from Tianjin Damao Chemical Reagent Factory. Jinmai 79 wheat seeds (*Triticum aestivum* L.) were purchased from the Institute of Wheat Research, Shanxi Academy of Agricultural Sciences. *S. aureus* was provided by the Microbiology Laboratory of Shanxi Normal University. HeLa cells were provided by the A TCC Cell Conservation Center.

### 2.3. Preparation of C-CDs

C-CDs were synthesized by a one-step hydrothermal method. Briefly, 0.2 g of pulverized coal powder (below 200 mesh) was added to a round bottom flask with 20 mL of H_2_O_2_ at 30% mass fraction and 10 mL of ultrapure water, placed on a magnetic heating stirrer, and stirred continuously at 70 °C to fully react with the coal powder for 4 h [18]. A brown liquid was obtained by natural cooling to room temperature, and the brown supernatant was obtained by centrifugation at 4000 r/min for 30 min in a high-speed centrifuge. The brown supernatant was filtered through a 0.22 μm membrane and dialyzed using a dialysis membrane with MWCO:1000 molecular retention capacity for 24 h. Finally, the aqueous solution of C-CDs after dialysis was collected and freeze-dried to obtain the final brown solid powder.

### 2.4. Biostress of C-CDs

In this study, we investigated the biotoxic effects of C-CDs on wheat, *S. aureus*, and HeLa cells using coal-based carbon-dotted nanomaterials as stress factors. Wheat was grown in an artificial climate incubator (RGX-280F) at 25 °C, and the effects of C-CDs on wheat germination rate, biomass, photosynthesis, superoxide dismutase activity, catalase activity, ATP production, and other indices were determined; *S. aureus* was cultured in a bifunctional air-bath thermostatic shaker (ZD-85) in a survival environment of 37 °C. Briefly, 24-well plates containing *S. aureus* were spiked with different concentrations of C-CDs and irradiated with 365 nm light for 1 min. Then, *S. aureus* was cultured by spreading it uniformly in LB medium, and colony numbers were observed as a function of C-CDs; HeLa cells were cultured in a constant temperature incubator (MCO-15AC) at 37 °C in a 5% CO_2_ environment for C-CD toxicity experiments. Briefly, HeLa cells were first incubated with different concentrations of C-CDs for 4 h and then irradiated with UV light at 365 nm at different times. Finally, the irradiated HeLa cells were incubated in a nutrient solution without C-CDs, and the survival rate of the cells was calculated.

## 3. Results

### 3.1. Characterization of C-CDs

**Figure 2:**
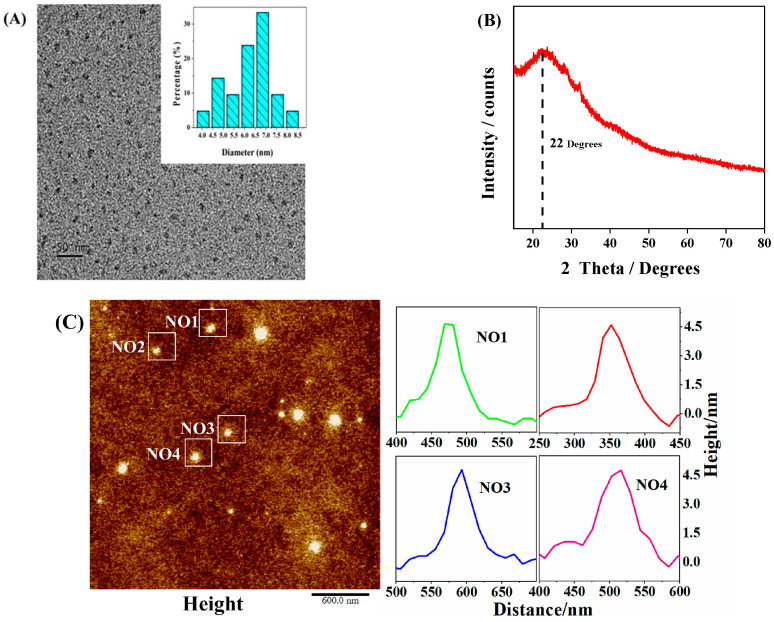
Morphological and dimensional characterization of C-CDs: (**A**) TEM image of C-CDs (inset is particle size distribution); (**B**) XRD image of C-CDs; (**C**) AFM image of C-CDs.

As shown in Figure 2A, the C-CDs are well dispersed and relatively uniform, with an average particle size of about 4.6 nm and no noticeable lattice stripes, which may be a polymeric structure. X-ray diffraction (XRD) patterns (Figure 2B) show a peak at 22 Theta degrees, indicating a highly disordered carbon atom arrangement, probably due to the polymerization reaction generating a co-bonded cross-linked backbone, with a partial order formed within the C-CDs [24], confirming the results observed by TEM. In this study, the three-dimensional morphology of the sample surface was also characterized by atomic force microscopy (AFM) (Figure 2C), and since the high and low undulating states of the sample surface can be obtained numerically and also displayed in a rich three-dimensional simulation of the sample morphology [25,26], it can be known that the height of the C-CDs is between 4–5 nm, which is in general agreement with the TEM results.

**Figure 3:**
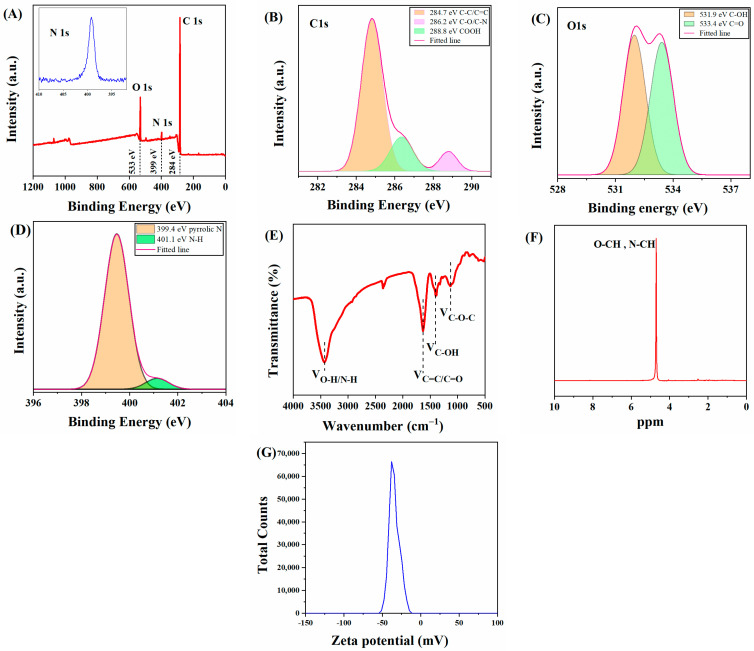
Characterization of functional groups contained in C-CDs: (**A**) XPS image of C-CDs; (**B**) C1s, (**C**) O1s, (**D**) N1s high-resolution spectra; (**E**) IR spectrum of C-CDs; (**F**) ^1^H nuclear magnetic resonance spectrum (NMR) of C-CDs; (**G**) Zeta potential map of C-CDs.

To further investigate the surface composition of C-CDs, X-ray diffraction spectroscopy (XPS) and Fourier transform infrared spectroscopy (FT-IR) tests were performed on C-CDs in this study. The full spectrum of C-CDs (Figure 3A) shows that C-CDs are mainly present in C, N, and O elements, corresponding to three characteristic peaks at 284 eV, 399 eV, and 533 eV. Figure 3B shows the high-resolution C1s spectrum of C-CDs, which can be decomposed into three characteristic peaks of C-C/C=C (284.7 eV), C-N/C-O (286.2 eV), and COOH (288.8 eV) by fitting analysis. Figure 3C shows the high-resolution energy spectrum of O1s, which offers two characteristic peaks at C-OH (531.9 eV) and C=O (533.4 eV). The high-resolution energy spectrum of N1s shows two characteristic peaks at 399.4 eV and 401.1 eV for the pyrrole-N and N-H bonds, respectively (Figure 3D). From the FTIR analysis of the C-CDs (Figure 3E), it can be seen that the broad peak at 3430 cm^−1^ indicates the presence of -OH and N-H; 2942.1 cm^−1^ is a stretching vibration peak of C-H; the peak at 1631 cm^−1^ is probably a stretching vibration peak of C=C/C=N; 1400 cm^−1^ corresponds to a stretching vibration peak of C-OH; and 1130 cm^−1^ has an absorption vibration peak corresponding to the C-O-C stretching vibration. The FTIR analysis results further indicate that the surface of the C-CDs contains carboxyl groups, hydrocarbon groups, and nitrogen-containing groups and that the surface of the generated C-CDs has good water solubility due to the oxidation of coal by hydrogen peroxide with oxygen-containing functional groups. To analyze the hybridization mode of C-CDs, nuclear magnetic resonance (NMR) hydrogen spectroscopy was performed using deuterated water (D_2_O) as the solvent. In Figure 3F, the characteristic peaks between 4–6 ppm are streptavidin and sp^2^-hybridized carbon atoms. The −ζ test (Figure 3G) results indicate that the surface potential of C-CDs is −37.9 mV, which may be due to the more abundant surface carboxyl groups [27].

**Figure 4:**
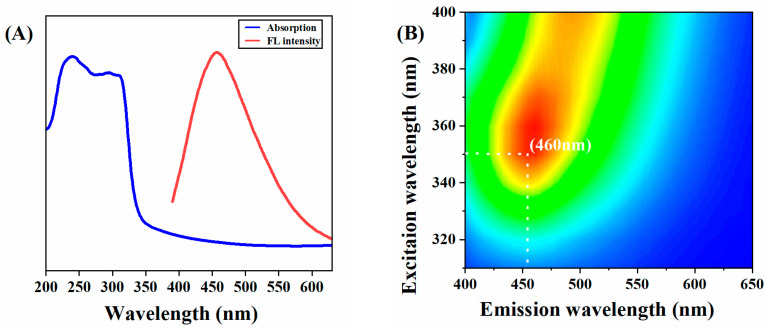
Fluorescent properties of C-CD: (**A**) C-CD UV absorption spectrum and fluorescence emission spectrum; (**B**) The 3D fluorescence spectrum of C-CDs. (Color depth represents fluorescence intensity).

In this study, a series of optical characterizations of C-CDs was carried out. As shown in Figure 4A, the UV-vis absorption of C-CDs is located at 310 nm, which can be attributed to the n-π* jump of the conjugated C=O/C=N bond. There is a significant overlap between the excitation and emission spectra. It can be inferred that the conjugated bond (C=O/C=N) at 310 nm is the origin of the photoluminescence. From Figure 4B, it can be seen that the best excitation peak of C-CDs in aqueous solution is λ_ex_ = 350 nm, and the best emission peak is λ_em_ = 460 nm. Since C-CDs have no prominent lattice structure, the surface state and molecular state are the main reasons for their fluorescence emission, and it can be inferred that the fluorescence emission is obtained through surface state modulation. The fluorescence emission mechanism is that the surface of the C-CDs is rich in carboxyl and hydroxyl groups, and the structural distortion of conjugated carbon atoms reduces the band gap of the C-CD’s energy level, resulting in the observable fluorescence emission with the excitation-dependent phenomenon.

The above results indicate that negatively charged C-CDs with abundant oxygen- and nitrogen-containing functional groups on the surface and good water solubility with unique photocatalytic properties were synthesized in this study.

### 3.2. Toxic Effects of C-CDs on Wheat

**Figure 5:**
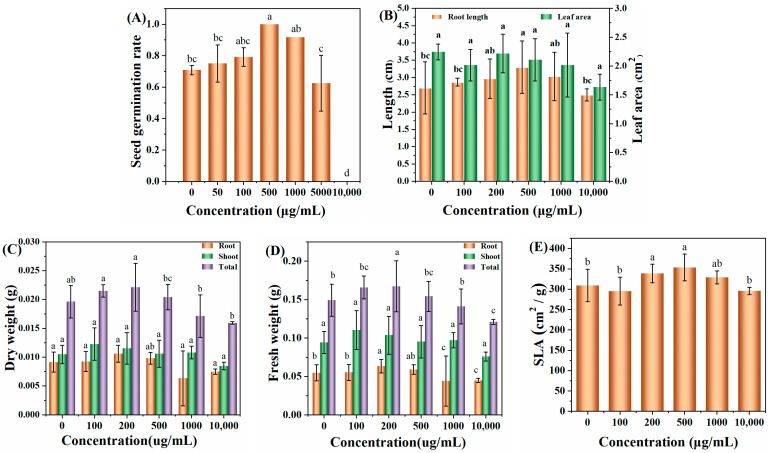
Effects of hydroponic cultivation with different concentrations of C-CDs on wheat phenotypes: (**A**) the effect of C-CD aqueous solution culture on the germination rate of wheat seeds; (**B**) effect of C-CDs on wheat seedling root length and effect of leaf area; (**C**) effect of different concentrations of C-CD nutrient solution on leaf dry weight, root dry weight, and dry biomass of wheat seedlings; (**D**) different concentrations of C-CD nutrient solution on wheat seedlings leaf fresh weight, root dry weight, fresh weight, and fresh biomass; (**E**) Effects of different concentrations of C-CD nutrient solution on specific leaf area of wheat seedlings. Different letters indicate significant differences.

Figure 5A shows the effect of different concentrations of C-CD aqueous solution (0, 50, 100, 500, 1000, 5000, and 10,000 μg/mL) on the germination rate of wheat seeds soaked for three days; it can be seen that without the addition of C-CDs, the germination rate of wheat seeds incubated for three days was 71.6%; with the increase in C-CD concentration, the germination rate of wheat seeds gradually increased; when the concentration of C-CDs increased again, the germination rate of wheat seeds incubated for three days could reach 98%. The germination rate of wheat seeds could achieve 98% at 500 μg/mL C-CDs for three days. When the concentration of C-CDs was increased again, the germination rate of wheat seeds gradually decreased to zero at 10,000 μg/mL, indicating that the low concentration of C-CDs had a promoting effect on the germination of wheat seeds. In contrast, the high concentration of C-CDs had an inhibiting effect on the germination of wheat seeds. The reason may be that at low concentrations, C-CDs promoted the water absorption process of seeds due to their water absorption properties, increasing germination rate. In contrast, at high concentrations, C-CDs were densely adsorbed on the seed surface, hindering the normal respiration of seeds and leading to a significant decrease in germination rate.

In addition, when the concentration of C-CDs was 200 μg/mL to 500 μg/mL, the photosynthetic rate, root length (Figure 5B), dry weight (Figure 5C), fresh weight (Figure 5D), and specific leaf area (Figure 5E) of wheat seedlings increased to different degrees. When the concentration of C-CDs exceeded 500 μg/mL, the root length, fresh weight, dry weight, and specific leaf area indices of wheat gradually decreased.

**Figure 6:**
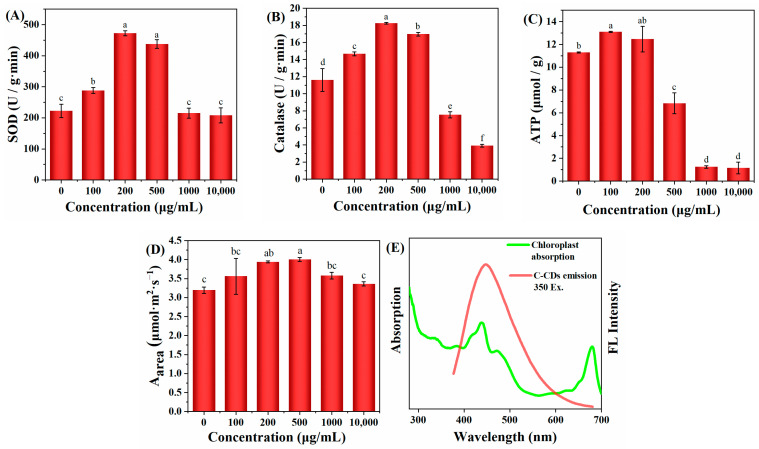
Effects of different concentrations of C-CDs in hydroponics on physiological indices of wheat: (**A**) effects of different concentrations of C-CD nutrient solution on superoxide dismutase activity; (**B**) effects of different concentrations of C-CD nutrient solution on catalase activity; (**C**) effects of different concentrations of C-CD nutrient solution on ATP production; (**D**) the effect of C-CD aqueous solution culture on the photosynthetic rate per unit area of wheat seedlings; (**E**) chloroplast absorption spectrum (green) and emission spectrum of C-CDs (red). Different letters indicate significant differences.

To elucidate the mechanism of changes in physiological and ecological indicators of wheat seedlings, this study further investigated the effect of C-CD culture on wheat seedlings by measuring the activity of superoxide dismutase (SOD) by the nitrogen blue tetrazolium photo-oxidation reduction method; the primary function of SOD is to catalyze the dismutation of superoxide anion radicals to H_2_O_2_ and O_2_ and reduce the cytotoxicity of superoxide anion radicals. As shown in Figure 6A, 200–500 μg/mL C-CDs were able to increase the SOD activity of wheat seedlings significantly (*p* < 0.05) and promote plant metabolism. In addition, intracellular catalase (CAT) could further catalyze the decomposition of H_2_O_2_ into O_2_ and H_2_O. Under adversity stress, H_2_O_2_ is easily produced in cells to destabilize the membrane system, while CAT could decompose H_2_O_2_ into H_2_O and O_2_, thus maintaining the membrane stability. The above results suggest that low concentrations of C-CDs may enhance the resistance of wheat seedlings to stress.

When a photosynthetic photoreaction occurs, the generated electrons are transferred to PS and PS I via the electron transport chain. In PS I, the excited electrons are transported to the electron transport chain, after which the electrons can be transferred back to the electron transport chain in PS II, thus providing a circular pathway for ATP production, or the electrons can be used to reduce NADP+ to NADPH. As shown in Figure 6C, wheat seedlings cultured with low concentrations of C-CDs had significantly increased ATP production (*p* < 0.05), but the higher concentration (500 μg/mL) of C-CDs decreased ATP production. The reduced ATP production could not provide enough “assimilation power” for the dark reaction of photosynthesis, making the plant less able to fix CO_2_ and unable to accumulate more biomass, which is consistent with the changes of biomass in Figure 5C,D. In addition, from Figure 6E, it can be seen that C-CDs capture part of the light energy (mainly UV light) into the spectrum used by wheat seedlings, which can increase the photosynthetic rate of wheat seedlings (Figure 6D).

The above results showed that under hydroponic conditions, low concentrations of C-CDs promoted the germination rate and growth of wheat, and high concentrations of C-CDs inhibited the germination rate and growth of wheat, indicating that higher concentrations of C-CDs had toxic effects on wheat seeds and roots.

### 3.3. Toxic Effects of C-CDs on Bacteria

**Figure 7:**
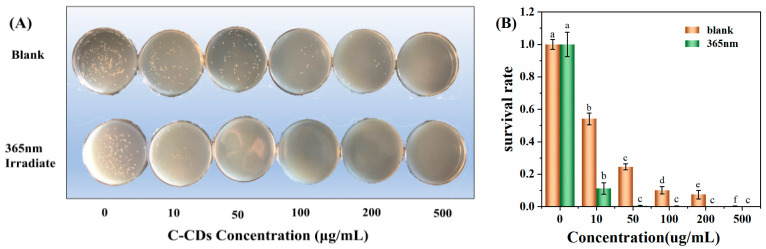

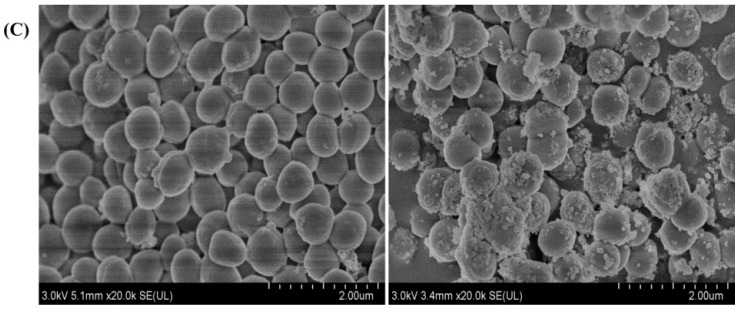
Effects of different concentrations of C-CDs on *S. aureus*: (**A**) inhibition of *S. aureus* by different concentrations of C-CDs under no light and 365 nm irradiation; (**B**) survival rate of *S. aureus* in the presence of different concentrations of C-CDs; (**C**) SEM images of *S. aureus* before (**left**) and after (**right**) with C-CDs. Different letters indicate significant differences.

The experiments were set up with different concentrations of C-CDs, and *S. aureus* was incubated for 5 min under dark conditions and 365 nm light conditions and then coated on LB medium for continued growth. As shown in Figure 7A, with the increase in C-CD concentration, C-CDs had a significant inhibitory effect on *S. aureus* with or without light irradiation. The inhibition intensity was further enhanced under 365 nm light source irradiation, and the inhibition rate reached 98.6% at 50 μg/mL (Figure 7B). The above results indicated that C-CDs inhibited *S. aureus* at specific concentrations, and the inhibitory effect was more substantial under 365 nm light. This study further investigated the reasons for C-CDs inhibiting *S. aureus*. From the changes in the SEM morphology of bacteria before and after treatment (Figure 7C), it is clear that after being treated with 50 μg/mL C-CDs for 5 min, a large number of C-CDs were attached to the surface of bacterial cells, which may lead to the blockage of bacterial cell membranes, making it difficult for material exchange and energy flow inside the bacterial cells with the outside world.

### 3.4. Evaluation of the Toxicity of C-CDs on Eukaryotic Cells

**Figure 8:**
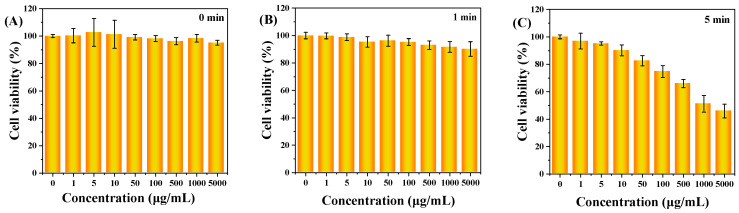

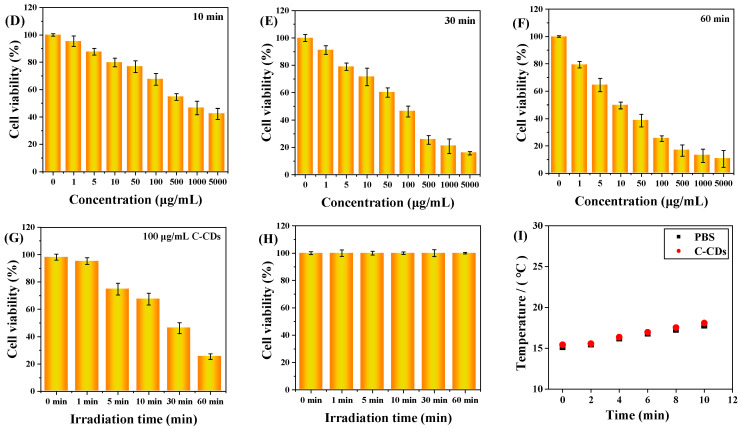
The effect of C-CDs on the activity of HeLa cells in vitro. MTT assay: (**A**) Under no-light conditions, different concentrations of C-CDs (0, 1, 5, 10, 50, 100, 500, 1000, 5000 μg/mL) on the activity of HeLa cells; (**B**) the effects of different concentrations of C-CDs (0, 1, 5, 10, 50, 100, 500, 1000, 5000 μg/mL) on the activity of HeLa cells after 1 min of illumination; (**C**) the effect of different concentrations of C-CDs (0, 1, 5, 10, 50, 100, 500, 1000, 5000 μg/mL) on the viability of HeLa cells after 5 min of illumination; (**D**) the effects of different concentrations of C-CDs (0, 1, 5, 10, 50, 100, 500, 1000, 5000 μg/mL) on the viability of HeLa cells after 10 min of illumination; (**E**) the effect of different concentrations of C-CDs (0, 1, 5, 10, 50, 100, 500, 1000, 5000 μg/mL) on the viability of HeLa cells after 30 min of light exposure; (**F**) the effect of different concentrations of C-CDs (0, 1, 5, 10, 50, 100, 500, 1000, 5000 μg/mL) on the activity of HeLa cells after 60 min of light; (**G**) the effect of different 365 nm irradiation times (0, 1, 5, 10, 30, 60 min) on the activity of HeLa cells (the concentration of C-CDs was 100 μg/mL); (**H**) the effect of light time (0, 1, 5, 10, 30, 60 min) on the activity of HeLa cells without additional C-CDs; (**I**) the photothermal effect of PBS and C-CDs.

This study determined and analyzed the nanotoxicity of C-CDs on HeLa cells using the MTT method. HeLa cells were first incubated with different concentrations of C-CDs for 4 h, then divided into no-light and 365 nm light groups, and the cells were treated with varying lengths of time and then replaced with fresh culture medium without C-CDs and continued to incubate for 24 h. Figure 8A shows the effect of different C-CD concentrations (0, 1, 5, 10, 50, 100, 500, 1000, 5000 μg/mL) on HeLa cell activity. The results show that even though the concentration of C-CDs had reached 5000 μg/mL, it still had almost no effect on cell viability. As the time of light irradiation increased, 1 min (Figure 8B), 5 min (Figure 8C), 10 min (Figure 8D), 30 min (Figure 8E), and 60 min (Figure 8F) showed a gradual decrease in cell activity; except for the insignificant effect of irradiation for 1 min on cell activity, the irradiation times from 5 to 60 min all significantly affected cell activity.

It was further observed that the survival rate of cells under 100 μg/mL C-CD conditions was 46.2% when the light time was 30 min and only 25% when the C-CDs concentration was 100 μg/mL for 60 min (Figure 8G). However, when no additional light was added, Figure 8A showed that different concentrations of C-CDs (0, 1, 5, 10, 50, 100, 500, 1000, 5000 μg/mL) had essentially no effect on HeLa cell activity while 365 nm light had essentially no effect on HeLa cell activity without the addition of C-CDs, as shown in Figure 8H. As can be seen from Figure 8I, the solution temperature was not any higher when 365 nm irradiation was performed in the presence of C-CDs compared to the PBS buffer. Thus, it was ruled out that C-CDs absorbed heat to damage HeLa cells. The above results suggest that the specific wavelength irradiation at 365 nm is the critical condition leading to C-CDs’ toxicity to cells.

**Figure 9:**
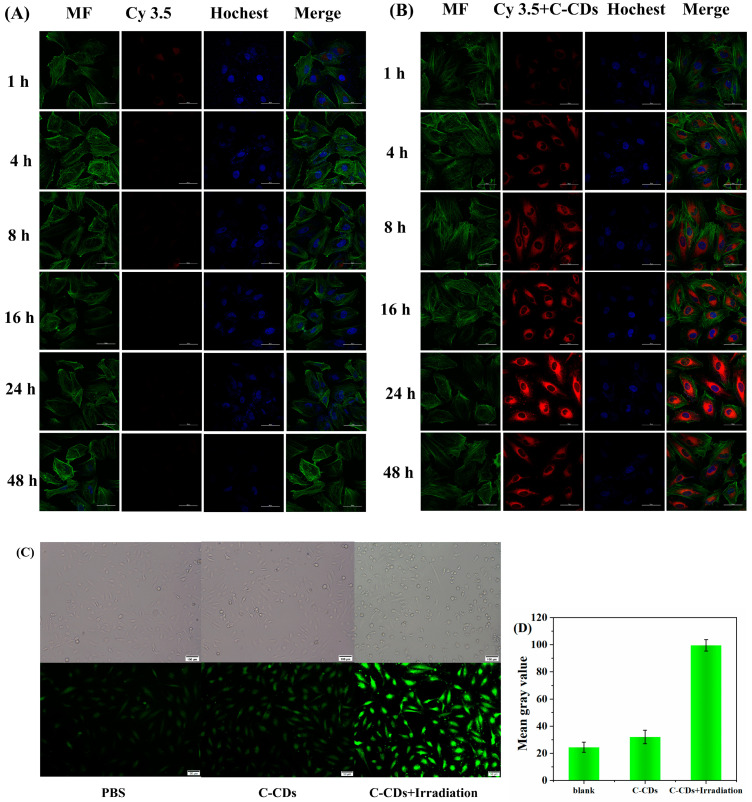
Cellular uptake of C-CDs: HeLa cells were incubated with Cy3.5 (**A**) and Cy3.5-labeled C-CDs (**B**) For 1 h, 4 h, 8 h, 16 h, 24 h, and 48 h under confocal microscopy. Bottom image, cell filaments (MF) were stained with phalloidin in green, and nuclei were stained with Hochest 33342 in blue, scale bar is 50 µm; (**C**) DCFH-DA probe was used to label PBS and the generation of intracellular ROS after 365 nm LED illumination after C-CDs, scale bar = 50 µm; (**D**) green light grayscale intensity of HeLa cells.

To further investigate the mechanism of cytotoxicity of C-CDs, Cy3.5-labeled C-CDs were selected, and the behavior of nanoparticles in cells was examined by laser confocal fluorescence scanning microscopy (CLSM). Figure 9A represents the process of Cy3.5 (red fluorescent dye) in HeLa cells; it can be observed that almost no dye enters into the interior of the cell. Figure 9B demonstrates the results of C-CDs combined with Cy3.5 fluorescent dye together into the interior of the HeLa cells; a large amount of red fluorescence in the interior of the cell can be observed, which shows that the C-CDs have an excellent cell affinity. Incubation of HeLa cells with free Cy3.5 dye showed only weak fluorescence intensity from 1 h to 24 h, and the fluorescence intensity became extremely weak after 24 h; in comparison, the fluorescence intensity of the C-CDs-Cy3.5 group continued to increase from 1 to 24 h, and the fluorescence image reached the brightest at 24 h. The fluorescence intensity gradually decreased from 24 h to 48 h, probably because most of the C-CDs-Cy3.5 was metabolized by living cells. Figure 9C shows cells treated with 200 μg/mL of C-CDs under the irradiation of a 365 nm light source and stained with 2,7-dichlorodihydrofluorescein diacetate (DCFH-DA), which is a dye that can freely pass through the cell membrane and is hydrolyzed by intracellular esterases to produce DCFH after entering the cell. In the presence of reactive oxygen species, DCFH oxidized to produce the fluorescent substance DCF, and the intensity of the green fluorescence was proportional to the intracellular reactive oxygen species level, which was used to measure the production of intracellular ROS. DCFH-DA was able to react with intracellularly generated ^1^O_2_, showing a green fluorescence signal. Compared with the blank group, the irradiation of C-CDs + 365 nm light source caused a large amount of ^1^O_2_ to be generated in the cells, leading to cell death (Figure 9D).

### 3.5. Mechanism of Phototoxicity of C-CDs

**Figure 10:**
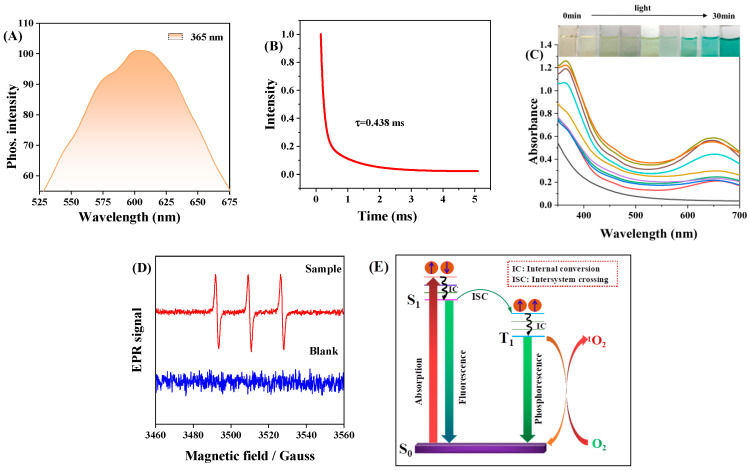
(**A**) Phosphorescence spectra of C-CDs under 365 nm light excitation; (**B**) phosphorescence lifetime of C-CDs; (**C**) absorption spectra of C-CD aqueous solution with added TMB under 365 nm light irradiation with time; (**D**) EPR spectra of 10 μL TEMP (10M) mixed with 2 μL C-CDs (10 mg/mL) under 365 nm light irradiation (TEMP is a unique spin trap of ^1^O_2_); (**E**) C-CD fluorescence and phosphorescence and O_2_ to ^1^O_2_ energy transfer process.

To elucidate the reason for the strong biotoxicity of C-CDs to *S. aureus* and HeLa cells under light exposure, this study was conducted with 365 nm light. Because of the similar chemical origin of phosphorescence and photosensitization, ^1^O_2_ is often associated with the excited trilinear state; therefore, the solid phosphorescence properties of C-CDs were first investigated, and the optimal phosphorescence emission of C-CDs was found to be 600 nm from Figure 10A. The phosphorescence lifetime of C-CDs was 438 μs (Figure 10B), allowing us to infer that the generation of ROS by C-CDs might be related to phosphorescence.

In order to investigate the photocatalytic activity of C-CDs, this study investigated the catalytic oxidation performance of C-CDs using 3,3′,5,5′-tetramethylbenzidine (TMB) as a peroxidase substrate. When C-CDs were added, TMB was oxidized under the irradiation of 365 nm light source, and a significant color reaction occurred (Figure 10C). Electron paramagnetic resonance (EPR) spectral analysis (Figure 10D) indicated that C-CDs produced ^1^O_2_ under light irradiation. It was ^1^O_2_ that oxidized TMB to change color.

Since the triplet-state exciton leap generates C-CD phosphorescence (RTP), RTP photosensitizers have a higher quantum yield of T1-state excitons. In contrast, the ground state of O_2_ is a triplet state, so RTP photosensitizers can generate ^1^O_2_ with higher oxidative activity through energy transfer of T1-state excitons with oxygen molecules (Figure 10E). Therefore, C-CDs have a more significant toxic effect under light, which is more evident in HeLa cell experiments (Figure 8).

## 4. Discussion and Conclusions

The results of the above studies show that the effects of C-CDs on wheat seeds and seedlings are mainly manifested at low concentrations to promote growth and high concentrations to inhibit growth. Some studies have shown that the nanomaterials can improve the water absorption capacity of seeds through up-regulation of the expression of water channel protein genes to promote the germination rate of seeds [28] and that the increase in the germination rate of seeds with a low concentration of C-CDs may be due to the incorporation of C-CDs to enhance the water uptake rate of the seeds [29]. A low concentration of C-CDs promoted the increase in photosynthetic rate per unit area of wheat seedlings, which led to the increase in fresh weight, dry weight, and other plant growth indices, which was mainly due to the ability of C-CDs to capture a portion of the UV light energy that can be converted into the spectrum used for photosynthesis, and thus increased the photosynthetic rate. However, high concentrations of C-CDs adsorbed densely on the surface of the seeds or on the surface of the leaves hindered the normal respiration of the seeds or blocked the stomata of the leaves and the passage of sunlight, resulting in a significant decrease in the germination rate and photosynthesis. The toxicity of C-CDs to bacteria and HeLa cells was mainly manifested as a phototoxicity-enhancing effect, which was mainly due to the fact that C-CDs were able to act as photosensitizers in the presence of light (365 nm) to convert O_2_ to produce oxidative toxicity, which in turn enhanced the toxic effect of C-CDs.

In this study, C-CDs nanomaterials were prepared from inexpensive raw coal from wellheads, and the toxic effects of C-CD nanomaterials on wheat were elucidated by investigating the relationship between the concentration of C-CDs and seed germination rate and seedling photosynthesis. In addition, this study also evaluated the nanotoxicity effects of C-CDs on bacteria (*S. aureus*) and animal cells (HeLa cells) and found that the toxicity of C-CDs to bacteria in bacterial experiments was divided into two main types: first, C-CDs attached to the surface of bacteria, causing bacterial cell membrane blockage and death; second, under specific light source (365 nm) irradiation, C-CDs produce more ^1^O_2_, which accelerates bacterial cell damage and apoptosis. The toxicity experiments found that C-CDs did not significantly inhibit the growth of HeLa cells at a concentration of 5000 μg/mL, and HeLa cells could phagocytose C-CDs. Still, under 365 nm irradiation, C-CDs produced a large amount of ^1^O_2_, which could lead to HeLa cell apoptosis at low concentrations. This study can provide a theoretical and practical basis for safely utilizing C-CDs in various fields.

## Data Availability

Data are contained within the article and supplementary materials.

## Supplementary Materials

The following supporting information can be downloaded at: https://doi.org/10.5281/zenodo.10223805 (accessed on 5 November 2023).
